# Policy and practice review consensus statements and clinical guidelines on managing pediatric trauma and orthopedics during the COVID-19 pandemic: a systematic review on the global response for future pandemics and public health crises

**DOI:** 10.3389/fped.2024.1453574

**Published:** 2024-10-01

**Authors:** Kapil Sugand, Chang Park, Arash Aframian, Chinmay M. Gupte, Khaled M. Sarraf, COVid Emergency Related Trauma and orthopaedics (COVERT) Collaborative

**Affiliations:** ^1^ MSK Lab, Imperial College, London, United Kingdom; ^2^ Department of Trauma & Orthopedics, St Mary’s Hospital, Imperial College Healthcare NHS Trust, London, United Kingdom; ^3^ Peripheral Nerve Injury Unit, Royal National Orthopaedic Hospital, Stanmore, United Kingdom

**Keywords:** COVID-19, pediatric orthopedics, consensus statement, guidelines, trauma, global response

## Abstract

**Introduction:**

The COVID-19 pandemic has been recognized as an unprecedented global health crisis. Over 7 million mortalities have been documented with many paediatric fatalities. Trauma and orthopaedic care, much like other specialities, were marginalized due to resource allocation during the pandemic which affected paediatric care. This is the first systematic review to centralise and compile the recommended published guidelines from professional bodies in principally English speaking countries on managing paediatric trauma and orthopaedic care. These guidelines will be required to be implemented sooner and more effectively in case of future pandemics with similar impact.

**Methods:**

A search was conducted on PubMed/MedLine, Cochrane Library and Embase using terms including p(a)ediatric or child* and/or COVID* or coronavirus or SARS-CoV-2 and/or trauma and/or orthop(a)edic* with a simplified MeSH heading [mh] in order to make the search as comprehensive as possible. General terminology was utilized to make the search as exhaustive as possible for this systematic review. Another search was conducted on resources available in the public domain from professional bodies publishing on consensus statements and clinical practice guidelines in countries where English is the principal language managing pediatric trauma and orthopedics. The review adhered to PRISMA guidance.

**Results:**

The search revealed a total of 62 results from both databases and professional bodies. Duplicates were removed. This was then reviewed to identify a total of 21 results which fit the inclusion criteria and included within the main analysis. The guidelines from professional bodies were outlined and categorized into aspects of clinical care.

**Discussion:**

The impact of COVID-19 pandemic has compelled for changes in clinical practice and pediatric management. The systematic review highlights the relevant guidelines on service provision for pediatric patients including indications for urgent referrals, surgical prioritization, reasons for follow-up and trauma guidelines. The rationale for care during the unpredictable evolution of the COVID-19 pandemic may have the potential to be translated and replicated in future pandemics of similar significance.

## Introduction

### Global impact

The COVID-19 pandemic was the greatest and most unprecedented era of global destabilization for healthcare services this century. The global mortality is over 7 million (1) of all confirmed cases. Although there has been much commented on the management of adult orthopedic trauma and elective patients, there has been less focus on the equally important issue of pediatric care for musculoskeletal disease and injury during this period. An estimated twenty million children are injured each year, with an incidence of one in every four children (2, 3). Furthermore, trauma is the leading cause of mortality and morbidity within the pediatric population while facing a concurrent decline in pediatric trauma programs (4). Consequently, challenges have risen when there is a burden of trauma in amidst a pandemic when limited resources and treatments have to be rationed.

### The global orthopedic response to the pandemic

Global orthopedic clinical practice has been substantially affected as a direct consequence of the pandemic like all other hospital specialities. Although there was suspension of elective orthopedic adult surgery, semi-elective and urgent elective pediatric care are still deemed to be essential. This pandemic led to firm governmental policies of social distancing and lockdown, which started in early 2020. Consequently, the pandemic forced the hand of governments, hospitals and official bodies from all over to address the growing concern of providing optimal orthopedic management for children.

### Aim

The aim was to curate, compile and summarize current literature on managing pediatric trauma and orthopedics during the COVID-19 pandemic. Relevant literature will be extracted and compiled into a systematic review from published studies as well as clinical guidelines and consensus statements dictated by professional bodies within principally English-speaking nations. The compilation outlined in this body of work will be able to direct global healthcare services to mobilize their public health resources more efficiently and quickly in case of future pandemics with similar impacts to COVID-19.

## Methods

**Figure 1:**
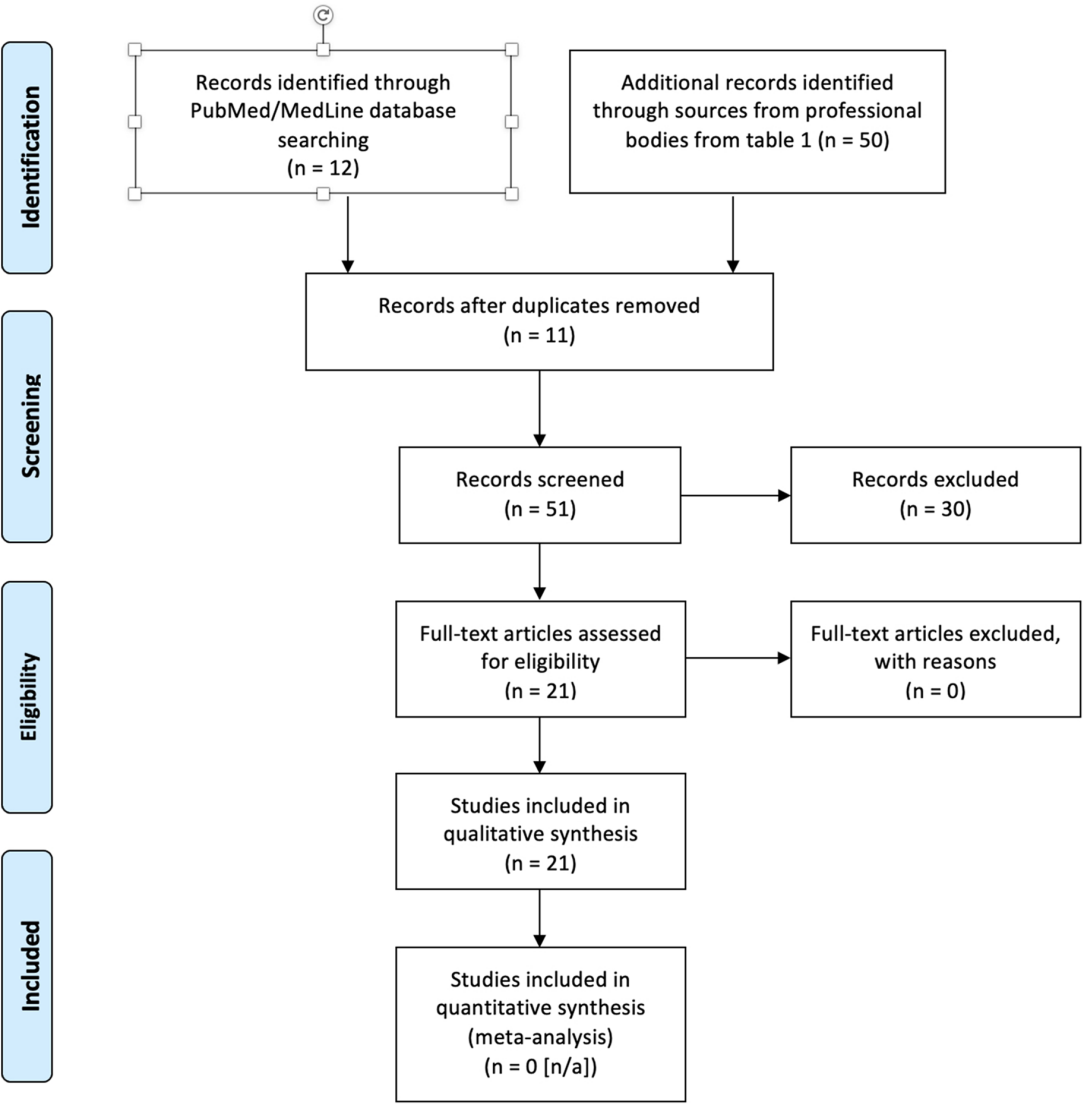
PRISMA diagram.

A systematic review was conducted on the clinical guidelines and consensus statements by professional bodies and research studies based on managing pediatric trauma and orthopedics during the COVID-19 pandemic. This was based on PRISMA reporting as seen in Figure 1. Published clinical guidelines and outcomes from any published research studies were screened by all authors (to avoid selection bias) for inclusion criteria prior to final analysis to compile a systematic review. A comprehensive search was conducted using PubMed/MedLine, Cochrane Library and Embase databases as well as from professional bodies specializing in pediatric orthopedic care from principally English-speaking countries. A collaboration between professional bodies within the UK, Europe, Australasia and North America has resulted in a compilation of guidelines to standardize and protocolize care on an international level with translational global applicability. The professional bodies consulted include the following as seen in Table 1.

**Table 1:** English-speaking professional bodies commenting on pediatric orthopedics.

Area	Professional body
USA	Orthopaedic Trauma Association (OTA) American College of Surgeons (ACS), American Academy of Orthopaedic Surgeons (AAOS) Centers for Disease Control and Prevention (CDC)
Canada	Canadian Orthopaedic Association (COA) Canadian Paediatric Orthopaedic Group (CPOG) Trauma Association of Canada (TAC)
North America	Paediatric Orthopaedic Society of North America (POSNA)
UK	National Health Service (NHS) England, NHS Improvement, National Institute for Care and Health Excellence (NICE), Royal Colleges of Surgeons (England, Edinburgh and Ireland), Cochrane Library, British Orthopaedic Association (BOA), NHS Greater Glasgow and Clyde (GC&C), British Society for Children's Orthopaedic Surgery (BSCOS)
Europe	European Paediatric Orthopaedic Society (EPOS) European Reference Network on rare bone diseases (ERN BOND) Italian Society of Pediatric Orthopaedics and Traumatology (SITOP)
Australia	Australian Orthopaedic Association (AOA), Australian Paediatric Orthopaedic Society (APOS) Australian Orthopaedic Trauma Society (AOTS)
New Zealand	New Zealand Orthopaedic Association (NZOA)
Global	World Health Organization (WHO) International Hip Dysplasia Institute Pandemic Work Group (IHDIPWG)

### Timeframe

1st January 2020 to 1st March 2021.

### Inclusion criteria

Only publications related to pediatric care mentioned within the literature (without a specified age cut-off) were included, as well as publications, consensus statements and clinical practice guidelines looking at care or management involving trauma and orthopedics. All publications were in English and guidelines were published from professional bodies commenting on pediatric surgical care. There were no restrictions on range of dates.

The search was expanded on resources available in the public domain from professional bodies publishing on consensus statements and clinical practice guidelines in countries where English is the principal language (including North America, the UK and Australasia) on managing pediatric trauma and orthopedics. Topics revolving around surgery, trauma, musculoskeletal, children and emergency medicine were screened.

### Exclusion criteria

Adult, non-orthopedic trauma, and non-orthopedic management. Not written in English. Not published either on PubMed/MedLine or on behalf of a professional body commenting on pediatric orthopedic care.

### Search terms

Medical Subject Headings (MeSH) terms used on PubMed/MedLine database included: P(a)ediatric or child* and/or COVID* or coronavirus and/or trauma and/or orthop(a)edics and *guideline* or *statement* or *consensus* with a simplified MeSH heading [mh] in order to make the search as comprehensive as possible.

### Ethics

This is a systematic review on information available within the public domain, hence there was no requirement for ethical approval.

## Results and discussion

### Disclaimer

The outlined figures were directly associated with protocols instigated during the COVID-19 pandemic. Most services, if not all, have resumed to their pre-pandemic quality and extent of provision. This historical review of guidelines developed for the COVID-19 pandemic may act as a proposal for guidelines should another pandemic arise in the future.

### Provision of urgent services during the pandemic

Due to the pressures on the health services, ability to provide routine elective care during this period has been severely limited. Hence, patients awaiting elective surgery are unlikely to be treated promptly, with the possibility that some may deteriorate in the meantime. Some conditions, however, are time critical and should be prioritized for urgent management. Furthermore, ongoing treatment may continue as much as resources allow but new treatments ought to be delayed as much as possible until specialist services resume to routine practice. Urgency of procedures have been categorized according to their level of clinical priority, as seen in Table 2 (5).

**Table 2:** Provision of urgent pediatric orthopedic services recommended by the UK NHS (5).

Condition	Recommendation
Existing patients	• Time-dependent conditions that require review as soon as local resources become available include: − Developmental Dislocation of the Hip (*DDH*): suspected cases/those currently undergoing harness/plaster immobilization − Congenital Talipes EquinoVarus (*CTEV*): currently undergoing cast treatment − Limb length discrepancy/malalignment—already undergoing treatment • Postpone non-essential long-term follow-up until situation improves for: − all other patients previously referred to pediatric orthopedic clinics − all patients under routine pediatric orthopedic clinic review
Inpatient management	• The majority of pediatric patients can be managed with day-case or short-stay surgery and provision should be optimized to minimize length of stay

### Service provision and management of CTEV, DDH and spinal pathologies during COVID-19

#### Congenital talipes equino Varus (CTEV)

BSCOS have published guidelines on managing CTEV (with the effective role of Ponseti casting) during the COVID pandemic (6) in line with Tables 2, 3, 6. Even though the advice is not to start new treatments during the pandemic (6), phase 2 of the pandemic allow for treatment to start as a time-urgent intervention (7). The UK Clubfoot Consensus Group suggest that there is good evidence that late treatment can still lead to good results; but treatment protocols for older babies and children can be more challenging (6). Successful casting is possible for those over 3 months but only with strict attention and to detail (8).

**Table 3:** UK BSCOS guidelines on recommended services for managing CTEV during the pandemic (6, 7, 9).

Service	Comment
Provision of services	• Plaster room (or equivalent) is required and consider a separate space for cast removal. • Consider alternative “non acute” venues for casting which may be more accessible and less anxiety provoking for families. • Only one family in a room at one time. • Appropriate use of waiting room spaces to maintain social distancing. • PPE requirements and availability need to be adequate for the duration of care, as do sanitizing facilities and consumables. • Theatre capacity remains limited and is presently being designated to more urgent conditions • If a large backlog exists or there are issues with hospital space and clinic times, the accelerated protocols can be safely used (11).
Treatment	• Ponseti management requires a minimum of 2 trained practitioners (with experience in managing treatment for babies over 3 months). • Early consideration should be given for the timing and location of the tenotomy procedure. • Tenotomies should not be delayed due to lack of opportunity. • Casting older babies with more underlying adipose tissue can be more challenging and without careful molding and application, casts may be more likely to slip. Policies need to be in place for the team to respond quickly and effectively to cast slips. • Older babies may be more challenging when performing a tenotomy under local anesthetic. • Older babies may be more challenging when introducing the foot abduction brace and settling them in to the orthosis.
Casting	• Plaster of Paris remains the material of choice. • Consider the potential need for the family to remove the cast at home if they are unable to attend appointments or cast slips. • The cast should not be removed at home, before the appointment, if it has stayed in a good position. • Soaking remains the preferred method of cast removal. • Families should be given guidance on how to determine if a cast has slipped and take a photograph before leaving the treatment room to enable subsequent comparison. Subsequent images can be communicated to the treating team remotely. • If a cast slips, it should be immediately removed by the family after appropriate advice. • If there is a lack of progress of correction after 5 casts and before 7 casts have been applied, help should be immediately sought from a high-volume specialist Ponseti service (12). A tenotomy should be pursued prior to discussion with that specialist service. • Attempting numerous casts with or without tenotomy in the face of multiple slipping casts or lack of progression causes complex deformities that can be more challenging to treat.
Tenotomy procedure	• The arrangements for tenotomies must be clarified before embarking on treatment. Where local provision is not possible, discussion with high-volume specialist sites is advised. • Some services perform tenotomies under local anesthesia in the theatre environment and some in the clinic. Both areas are subject to pressures depending on local activity. • Tenotomy under local anesthetic is advised to reduce anesthetic risk and pressures. Tenotomy under general anesthetic may be preferred in older babies.
First fitting of boots and bars	• This is better done ‘face to face’ due to the individual fitting needs and requirement to ensure parents are familiar with techniques. • If families are unable to attend for any reason, the fitting can be postponed by up to 2 weeks. The cast should remain in position until the brace can be fitted. • If remote fitting is planned, careful instructions on cast removal and boot fitting should be given. A secure telephone or video link may be available. • Arrangements for subsequent review and/or supply of larger boots and management of the bar width must be clarified with families at this stage.
Prioritization during Phase 2	• Infants who had treatment stalled in Phase 1, including those due first fitting of foot abduction brace • Infants with clubfoot awaiting primary treatment with casting • Families who are known to be struggling with the foot abduction brace at risk of recurrence • Children with pain or skin problems due to the brace • Children with painful feet who have completed Ponseti treatment.

Yet, delayed presentation or recurrent deformities have not been allowed treatment during phase 2. Guidelines on how to safely remove it without the need of a saw (9) have been published by BSCOS, as BOA has also produced guidelines on casting practice during the outbreak (10). Services to be provided for CTEV management during the pandemic have been outlined in Table 3.

#### Developmental dislocation of the hip (DDH)

##### Role of scanning in DDH

BSCOS released a set of guidelines (as a draft) on the role of imaging in DDH (13). Babies with screen positive results on the newborn physical examination should be referred for hip ultrasound. Due to the pressures on the current services, babies could receive the ultrasound prior to discharge from the hospital, and if normal, they can be discharged. If the hip ultrasound is not normal, the child should be rescanned when 6 weeks old if possible or as soon as routine services resume. Those with presence of DDH risk factors (i.e., breech and family history) who would usually have a hip scan at 6 weeks of age, should not be referred for scanning during the pandemic. This should be delayed until normal services resume for clinical examination and/or ultrasound/radiographic follow-up.

##### Management

Management is age sensitive but it is neither an emergency nor an elective issue (14). Although the provision of urgent services are still in place to manage DDH, primary and secondary surgical stabilization (which has not been clearly defined) are recommended to be delayed for months. Stabilization is considered on the assumption that harness treatment has failed but the effectiveness of harness or brace treatment (15) has been observed for infants less than 6 months of age.

The International Hip Dysplasia Institute Pandemic Work Group (IHDIPWG) supports the use of conventional treatment first especially due to service limitations and treating DDH should be secondary to saving lives endangered by the pandemic (14). Data from the International Hip Dysplasia Registry (IHDR) has also observed 91% success for closed reduction (16) in infants with a median age of 8 months (range 1–20). These results reassure both patients and surgeons that delayed closed or open reduction may not preclude a successful functional result even in older infants. Nevertheless, a longer delay in definitive treatment of DDH results in more challenges to achieve a successful closed reduction. In turn, closed reduction may be circumvented in place for open technique.

The challenges with delayed treatment lasting for months are due to contractures as well as disease evolution of bony and tissue morphology. Nevertheless, management of DDH can often be postponed by several months without seriously compromising final results (14), thus buying the clinician more time to treat until it is safe. Special circumstances have been outlined by the IHDIPWG and the POSNA as seen in Table 4.

**Table 4:** Special circumstances for DDH treatment according to IHDIPWG and POSNA (14).

Age (weeks)	Special circumstances
0–6	If DDH diagnosis is made by a positive Barlow/Ortolani sign, treatment may be initiated with the Pavlik method (ideally by trained healthcare provider) without ultrasound if unavailable. Follow-up may be conducted by telecommunications for the clinician to evaluate harness fit and problems, whereby adjustments can be made under direct guidance. Ultrasound imaging may be performed less frequently while in the harness rather than the usual 1–2 week intervals.
6–24	Treatment is similar to that in the 0–6 week age group. Although benefit exists from commencing treatment sooner, the probability of successful conservative treatment depends more on severity of dislocation than on prompt initiation of treatment in this age group (15, 17). Modest delays in treatment are unlikely to substantially compromise the final outcome. If risk factors are present with a normal clinical exam, or if the examination for hip instability is uncertain, imaging can be delayed until resources are available. A delay in diagnosis is unlikely to substantially alter management or change the final outcome of treatment.
>24	If resources are limited, Pavlik harness treatment may be attempted in some circumstances. However, closed reduction with serial cast management, or surgical open reduction procedures can be postponed for several months without substantially compromising results prior to 18 months of age.

DDH, developmental dysplasia of the hip; IHDIPWG, International Hip Dysplasia Institute Pandemic Work Group; POSNA, Pediatric Orthopaedic Society of North America.

### Spinal pathology

There has been no definition of osteomyelitis (or infective discitis) involving the spine as an emergency in Table 5; but rather inflammatory spinal pain has been categorized as an urgent condition without mentioning sepsis as a cause. Inflammation may be caused by infection, trauma, autoimmune or rheumatological conditions that influence the timeframe and urgency of instigating different treatment options. In regards to deformity, AOA (19) classified pediatric scoliosis as category 2 for skeletally immature and with a curve equal or above 90 degrees, and as category 3 with curves less than 90 degrees.

**Table 5:** Pediatric referral guidelines for acute MSk pathology by the UK (18).

Condition	Recommendation
Prioritization of referrals	• Signs of non-accidental injury • Fever with/without systemic symptoms • Deteriorating pain, unresponsive to conservative management/medication • Escalating night pain that prevents sleep • Persistent joint stiffness with/without swelling with suspected synovitis
Emergency conditions	Conditions that are life or limb-threatening including: • *Non-accidental injury (NAI)*: those at risk, or are a victim, of abuse to be referred immediately to child protection services • *Infection (osteomyelitis/septic arthritis):* patients presenting with fever, a hot swollen painful joint with reduced range of movement and/or loss of function to be referred immediately. Special attention should be paid to hip examination where pain and restriction of internal rotation is a sensitive sign of any hip joint pathology, including sepsis.
Urgent conditions	The following warrants urgent referral: • *Slipped upper femoral epiphysis*: Any child with sudden onset leg pain and difficulty weight bearing. • *Malignancy*: Any child with escalating pain including night pain and new or worsening swelling. • *Acute inflammatory arthritis and suspected rheumatological conditions as follows* (abridged to conditions only rather than manifestations here): − Suspected juvenile arthritis − Suspected new-onset autoimmune connective tissue disease − Dermatomyositis − Suspected inflammatory spinal pain
Non-urgent conditions	The majority of MSk conditions that affect children are innocent, self-limiting or have treatable solutions, especially if symptoms are intermittent. Definitive management of the majority of these conditions will be possible even if delayed by 3–6 months and may therefore be postponed for the duration of the COVID-19 pandemic.

### Acute pediatric musculoskeletal (MSk) referrals during the COVID-19 pandemic

Most children infected with COVID-19 seem to develop sub-clinical symptoms or mount a milder immune response and illness. Hence, they are suspected to act as vectors to contribute to viral transmission. It is important to reduce both the number of hospital attendances and duration of clinical contact with children. The acute conditions and prioritization have been summarized in Table 5 (18) as follows:

#### Independent variables to acute referral guidelines

Although there are clear guidelines on pediatric referrals, this does still lend itself to certain independent variables that need to be addressed. These guidelines have also made an assumption that non-urgent conditions may be managed with delayed treatment of 3–6 months which has been echoed with a shorter timeframe of 2–4 months proposed by Farrell et al. (20) for chronic and developmental orthopedic conditions. The time delay will inevitably lead into wintertime without accounting for subsequent waves of the pandemic. There could have been further justification that “assuming that a point of resurgence of clinical care will be reached” within the proposed timeframe.

Conversely, SITOP (21) have outlined need for vigilance in the acute setting. It has recommended several indications for acute referrals for in-person clinical review. Those with an acute onset or progression of pain or functional impairment, especially in the absence of trauma, need to be reviewed in person to exclude sinister disease such as bone tumors, musculoskeletal infections (septic arthritis, osteomyelitis), acute rheumatic diseases (juvenile arthritis) or severe developmental diseases (for instance SCFE or Perthes disease), which may necessitate non-deferrable treatments.

#### Educational implications

There was concern of skills decay of residents in training due to the COVID-19 pandemic and this applies to all specialties. There has however been evidence to demonstrate the effectiveness of e-learning and modalities of simulation, which significantly improved preparedness, confidence, and comfort for residents with percutaneous closed reduction and pinning of a pediatric supracondylar humeral fracture (22). If the state of clinical practice remains this way, surgical bodies should be encouraging residents in seeking out alternative modalities for continuing their surgical training using simulation as adjuncts.

*Delayed presentations:* Another issue may be delayed presentation due to parental concerns of contracting COVID-19, in which case, there may be a trend towards delayed presentation to the hospital after a musculoskeletal injury or pain (this could be more concerning in the presence of infection or inflammation). Delayed presentation and missed pathology have commonly been observed in developing nations such as Malawi (23) due to limitations for both resources and patient access. Access to pediatric healthcare facilities are required to be constantly monitored and held accountable as the healthcare system will continue to succumb to increasing pressures due to the pandemic.

### Surgical prioritization in amidst the pandemic

Surgical prioritization has been proposed for patients requiring surgery during the COVID-19 pandemic (21, 24). This categorization assisted in planning the allocation of surgical resources. Surgical specialties will need to assess the needs of their patients and rely on surgical triaging when resources are stretched to prioritize their treatment in future pandemics much like what was executed during COVID-19. This will also include facilitating the development of regional surgical networks to sustain the delivery of surgery to those patients in need, in a timely fashion. For instance, one of the handful of pathologies that warrants emergency surgery within 24 h is septic arthritis. The devastating sequelae of pediatric septic arthritis has been supported by Swarup et al. (25) Conversely, SITOP have classified septic arthritis within their elective procedures to be managed arthroscopically within 4 weeks (21). The ACS has only dictated trauma with uncontrolled hemorrhage or penetration to be recognized as an emergency for pediatrics (26). Certain MSk pathologies have been identified for the pediatric population and classified in Table 6 as follows:

**Table 6:** Surgical prioritization guidelines recommended by the UK and Italian national bodies (21, 23, 24, 26–28).

Priority level		Criteria	Conditions
NHS 1 & SITOP	A	Emergency—operation needed < 24 h	• Septic arthritis/osteomyelitis • Fractures—Open/neurovascular compromise/skin compromise • Dislocated joints • Compartment syndrome • Limb-threatening injuries
	B	Urgent—operation needed < 72 h	• Slipped Upper Femoral Epiphysis (SUFE) • Multi-Disciplinary Team (MDT) directed suspected bone or soft tissue malignant tumors • Closed fractures—displaced, unreducible, unstable, articular/peri-articular/forearm/femoral • Exposed metalwork • Soft tissue injuries without severe neurovascular damage
NHS 2 *(SITOP priority A)*		Semi-elective surgery that can be deferred < 4 weeks	• MDT directed suspected, aggressive benign bone tumor • Biopsy for suspected or surgery for aggressive and malignant MSk tumours • Knee: locking, bucket handle meniscal tears, meniscal repair, loose bodies, osteochondral defects and fragments • Septic arthritis requiring arthroscopy^a^ • SUFE^a^ • Misdiagnosed, neglected and displaced fractures at follow-up • Hardware-related complication (e.g., infection) • Nerve injury or compression with recent onset palsy not responding to non-operative treatment
NHS 3 (SITOP priority B)		Semi-elective surgery that can be delayed < 3 months	• DDH—Primary joint stabilization • CTEV—Initial management including tenotomies • Limb length discrepancy/malalignment • Guided growth hardware removal in case of overcorrection • Ponseti method for CTEV in newborns (3–6 months) • Closed/open reduction and cast for CDH in newborns (3–6 months)
NHS 4		Elective surgery that can be delayed > 3 months	• DDH—secondary joint reconstruction • CTEV—late presenting/relapsed • Spasticity management • Corrective surgery for established deformity • Reconstruction for established joint instability (e.g., ACL/lateral ligament) • Metalware removal
SITOP priority C		Elective surgery that can be delayed < 6 months from start of post-peak period	• Minimally invasive surgery (percutaneous tenotomies, subtalar arthroereisis) • Elective arthroscopy due to degenerative pathology • Procedures that ought to be done at a definite range of age (e.g., epiphysiodesis, congenital joint dislocation prior to ambulation)
SITOP priority D		Elective surgery safely performed at end of pandemic	• Elective surgery in those skeletally mature • Limb lengthening • Osteotomy (pelvis and long bones) • Arthrodesis • Scoliosis surgery

NHS, National Health Service (UK); SITOP, Italian Society of Pediatric Orthopaedics and Traumatology.

^a^ Italian guidelines compared with British guidelines.

#### Limitations

Urgent operations were limited to forearm and femur fractures without accounting for other bones. This was a guideline which will be applicable to more than just long bones like the femur. It was up to the clinician to weigh up the risk and benefit of surgery during the COVID-19 outbreak in the best interest of the patient. The same principle would apply to defining primary stabilization of DDH which has not been specifically defined but left to the clinician's acumen. Managing Perthes disease has only been mentioned by SITOP (21).

#### Supporting guidelines from Australia on surgical prioritization

AOA (19) agreed that conditions in level 1A as well as infected fractures, and deep and extensively contaminated lacerations would require care within 4–12 h (i.e., priority 2) but may need treatment within 1–4 h (i.e., priority 1) if the patient is septic or in shock. AOA also urges more emergent timeframe (i.e., 12–24 h) for spica for extra-articular femoral and tibial fractures. However, AOA differs with the British guidelines on displaced articular fractures (e.g., supracondylar fractures) which is urged to be managed within 4–12 h in Australia as opposed to within 72 h in the UK.

The AOA defines category 2 as urgently needing treatment within 90 days. The conditions under this category cause pain, dysfunction or disability as well as unlikely to deteriorate quickly and unlikely to become an emergency. Examples include surgical treatment of postoperative complications (other than planned revision surgeries), and chronic and severe incapacitating pain which may include neurological deficiency with unambiguous findings on clinical examination and patient ability to function severely impacts activities of daily living to the extent that the patient could not manage their symptoms for a further 3–6 months. Category 3 is defined as elective surgery (delayed until the end of the pandemic) whereby treatment is needed at some point in the next year.

#### Supporting guidelines from Italy on surgical prioritization

SITOP (21) have devised a triaging system to guide their surgical prioritization which counts (i) duration of the pandemic period and the local epidemic density; (ii) availability and accessibility of hospitals and operating theatres; (iii) characteristics and severity of the pediatric orthopedic disease; (iv) range of age of patients which may be correlated with favorable surgical outcomes and even the feasibility of some procedures; and (v) type of operation and surgical technique (as some are at higher risk for transmission of infection).

If the patient or parents are known or suspected to be COVID 19-positive, the operation should be postponed until the COVID tests are negative which may take more than a month. Yet, if delaying surgery can seriously threaten the health of the patient, it is up to the surgeon to evaluate the urgency and safety of treatment. If results are absent in an urgent setting, then patients should be treated as if they have positive COVID results in the interim requiring full precautions and personal protection equipment by healthcare staff. Furthermore, surgical treatments belonging to the priority categories A (within 4 weeks) or B (within 3 months) should not be suspended during the pandemic especially interruption to routine services will be for more than 2–3 months. Hence, high priority interventions should be preserved, and ideally centralized in non-COVID hospitals (i.e., “clean sites”).

#### Elective surgery

There has been no set definition on the timeframe to classify “elective” surgery. Elective surgery is likely to be affected by clinical urgency, patient and healthcare worker safety, as well as conservation of healthcare resources (20, 21). It is worthwhile noting that a majority of mechanism of pediatric injuries will remain unaffected by social distancing since they occur within the home setting. Only SITOP (Italy) compiled a set of guidelines on elective surgery (Table 6), using groups A-D according to timeframes, which is mostly in line with the ones recommended by the British consensus (27). The major difference is the SITOP have categorized arthroscopic treatment of septic arthritis and managing SUFE within 4 weeks (category A) (21) which has been expedited by the NHS to category 1 (i.e., within 24 and 72 h respectively).

SITOP (21) has also commented on “post-peak periods” in which patients in categories B-C (i.e., 3 months from the start of the pandemic phase to within 6 months post-peak from the start of the pandemic) should be prioritized close to the end of the pandemic period. However, this would be dependent on the stage of outbreak, accessibility and availability of resources. Additionally, SITOP suggested that elective surgery post-peak period should be prioritized as those who (i) require minimally invasive, arthroscopic or percutaneous techniques; (ii) do not require post-operative intensive care unit recovery; (iii) can be safely managed as day-surgery or with minimal inpatient care (possibly < 3 days); (iv) have comorbidities should be postponed to late stage of the post-peak period (particularly cardiovascular or respiratory); and (v) must be performed within a definite range of age (for example guided growth procedures).

### Trauma guidelines for the principles and management of pediatric care

The BOA, the British orthopedic governing body, has been leading in the international reflection of current clinical practice in Trauma and Orthopedic surgery. The BOA released BOA Standards for Trauma (BOAST) guidelines specifically during the COVID-19 pandemic (27) to address ideal practice for various patient cohorts. So far, previous BOAST guidelines that refer specifically for children is the “Supracondylar Fractures in the Humerus in Children” (29). The management of supracondylar distal humerus fractures (as well as the commonest upper and lower limb fractures and joint injuries) during COVID-19 outbreak have been outlined in Scotland too (28). The new BOAST guidelines for managing pediatric MSk conditions have been outlined in Table 7.

**Table 7:** UK BOAST guidelines (26) during the COVID-19 outbreak.

	Pediatric care
Background	During the pandemic, there will be increased emphasis on managing children with non-op strategies and ↓ OP visits. The aim is to ↓ long-term consequences by prioritizing conditions that have immediate, permanent morbidity or lack a practical remedial option.
Principles	1. Always consider NAI. The principles of management are unchanged. 2. If necessary, the following suspected diagnoses may be managed without radiology at presentation: a. Soft tissue injuries. b. Wrist, forearm, clavicle and proximal humeral #. c. Long bone # with clinical deformity. d. Foot # without significant clinical deformity and swelling. 3. The following may be managed without a cast at presentation: a. Knee ligament and patellar injuries with bracing. b. Stable ankle # may with FAB/Softcast. c. Hindfoot/midfoot/forefoot injuries managed with FAB/plaster shoe. 4. A single F/U appt at 4–12 weeks is acceptable for the majority of injuries. Patient-initiated F/U is appropriate for the following injuries: a. Patellar subluxations + dislocations, knee ligament + meniscal injuries, excluding locked knees. b. Lateral malleolar # + suspected ankle avulsion #. c. Foot injuries, except suspected mid- and hindfoot injuries. d. Wrist, forearm, clavicle and humeral #, incl. proximal humerus. e. Gartland type 1& 2 supracondylar #
Management	Non-operative management 5. Many injuries may be definitively managed in a cast at presentation. Wherever possible, use reinforced Softcast for home removal: a. Extra-articular tibial # without neurovascular/soft tissue compromise. A small number of these patients may require intervention: • Admit if ↑ risk of CS (adolescent or high energy injuries). • Sedation for reduction of clinically important deformity. • Residual deformity or malunion may need corrective surgery. b. Displaced wrist # in those < 10 years. c. Undisplaced ankle + forearm #. d. Gartland types 1 + 2 supracondylar #
	Operative management Day-case surgery 6. Most may have it as a day-case: a. Reduced joint dislocations. b. # + abnormal neurology/soft tissue compromise resolving after treatment. c. Peri-articular #. d. Extra-articular femoral # in those < 6 years (spica cast). e. Displaced forearm #.
	Management of obligatory inpatients 7. A small number require IP treatment with anesthesia + surgery: a. Open # (consider wash out + windowed cast). b. Septic arthritis and osteomyelitis with subperiosteal collection. c. Femoral # in those > 6 years (operative stabilization). d. Displaced articular or peri-articular #, incl. Gartland type 3 supracondylar # and acute SUFE (7).

↑, increase/maximize; ↓, decrease/minimize/under; OP, outpatient; incl, including; ED, emergency department; F/U, follow-up; D/C, discharge; #, fracture; NAI, non-accidental injury; FAB, fixed ankle boot; CS, compartment syndrome; SUFE, slipped upper femoral epiphysis; appt, appointment.

SITOP strongly demands for pediatric orthopedic surgeons to review on a case-by-case and encourage non-operative treatments where possible (21). Parents should be taught on how to safely remove temporary and soft casts or splints to reduce need for follow-up. If surgery is required then the operations should be performed by experienced senior surgeons, preferably as day-cases, maximizing the use of closed reduction, percutaneous pinning, resorbable sutures and self-removable casts of splints to avoid need for in-person clinic follow-up.

Not relying on radiological resources can lend itself to missed fractures. Extent of “clinical deformity” and “swelling” are also open to subjectivity and non-conformity since their degree have not been explicitly defined. There have been no reports in the current literature that COVID-19 has mitigated the role of plain radiography in fracture diagnosis. Patients are still being treated according to best practice correlated resource availability. Day-surgery capacity ought to be maximized when there is no choice but to operate while conservative management and limiting outpatient visits should also be emphasized (20, 21). In fact, follow-up may be done utilizing tele-consultations and video-conferencing while imaging ought to be reserved only if it will change management.

European Reference Network on Rare Bone Diseases (ERN BOND), originating from 38 centers in 10 countries, has recommended increasing the dosage of vitamin D and calcium salts in children with various types of rickets as well as continuing with amino bisphosphonates in osteogenesis imperfecta (30). At least with correcting bone metabolism, the risk of fractures may be reduced and need for hospitalization and surgery minimized. The same expert network has also successfully set up a 24/7 telephone line “COVID-19 Helpline for Rare Bone Diseases” (30, 31) in order to open up channels of communication with healthcare professionals, patients and members of the public and provide specialist knowledge on managing individual needs.

### Patient injury pathways on follow-up

SITOP have vowed to monitor multicenter outcomes to ensure adherence to their recommendations (21). Monitoring is required to assess the safety and effectiveness of the recommendations and to estimate the impact of the coordinated initiative on the health of children with orthopedic diseases. It has been recommended to cancel all deferrable outpatient appointments except for those that cannot be postponed due to post-operative care (including removal of K-wires, cast removal or renewal and complex wound care). Regular follow-up visits should be temporarily suspended.

Clinical guidelines on managing orthopedic trauma have also been summarized by Scotland (28). Both non-operative and operative management options have been outlined depending other fracture configuration, extent of displacement and severity have been tabulated. There are also specific guidelines on following-up patient injury pathways during COVID-19 from Scotland as seen in Table 8.

**Table 8:** Follow-up guidance on patient injury pathways recommended by the UK (29).

Injury	Follow-up
Toddler fractures	Walking boot for 2 weeks and follow-up if not fully weight bearing
Limps	To be offered an opt-in letter and reviewed if not resolving
Metatarsal fractures	No follow-up requirement but offer appropriate advice leaflet and put into walking boot
Clavicle fractures	No follow-up requirement but offer appropriate advice leaflet
Buckle fractures	As per clavicle fractures
Wound reviews	If *no* respiratory symptoms and unable to reach family doctor for suture removal to be followed-up If respiratory symptoms present then to contact hospital for appropriate follow-up

### Future studies

Future studies may compare such guidelines between pediatric and adult populations. This could help in prioritizing hospital cases in case the adult and pediatric orthopaedic departments are united as one clinical secondary service. Trauma services offering appropriate management in particular to both populations will be complicated by unpredictable pressure during another pandemic. By that time, artificial intelligence may also play a role to justify and optimize cases according to severity, prognosis and outcome measures especially when limited resources during the pandemic will be required to rationed to benefit as many for as long.

## Conclusion

Every attempt and effort have been made to minimize the risk to the pediatric-patient population under trauma and orthopedics. There is still hypervigilance on non-accidental injuries as well as specific pathologies that warrant both emergent and urgent surgeries. This is the first systematic review on the publications, clinical guidelines and consensus statements from English-speaking professional bodies globally alongside research studies to be compiled on managing pediatric trauma and orthopedics during the COVID-19 pandemic. Pediatric care must continue to improve in the face of the pandemic to protect the health of the future generations and to stay alert in case of future pandemics with similar impacts.

## References

[1] World Health Organization. WHO Coronavirus Disease (COVID-19) Dashboard. Available online at: https://covid19.who.int (Accessed March 29, 2024).

[2] Danseco ER Miller TR Spicer RS. Incidence and costs of 1987–1994 childhood injuries: demographic breakdowns. Pediatrics. (2000) 105:E27. 10.1542/peds.105.2.e27 10654987

[3] Spady DW Saunders DL Schopflocher DP Svenson LW. Patterns of injury in children: a population-based approach. Pediatrics. (2004) 113(3 Pt 1):522–9. 10.1542/peds.113.3.522 14993544

[4] Tuason D Hohl JB Levicoff E Ward WT. Urban pediatric orthopaedic surgical practice audit: implications for the future of this subspecialty. J Bone Joint Surg Am. (2009) 91(12):2992–8. 10.2106/JBJS.H.01708 19952265

[5] NHS England and NHS Improvement. Specialty guides for patient management during the coronavirus pandemic. Clinical guide for the management of trauma and orthopedic patients during the coronavirus pandemic. Publications approval reference: 001559. April 14 2020 Version 2. Available online at: www.england.nhs.uk/coronavirus/wp-content/uploads/sites/52/2020/03/C0274-Specialty-guide-Orthopaedic-trauma-v2-14-April.pdf (Accessed March 16, 2024).

[6] British Society for Children’s Orthopaedic Surgery. UK Clubfoot Consensus Group Guidance on Management of Clubfoot During the COVID-19 Pandemic. Available online at: www.bscos.org.uk/covid19/resources.php?sldownload=Y292aWQvVUtDQ0cgQ2×1YmZvb3QgYW5kIENvdmlkLTE5LnBkZiwwLCwsLDEsMTU4Nzk5Mjc0MiwyY2FmY2QyOGZkNzFmOTVjMmVjNmRjODZkYjI2NzM3Nw%3D%3D/UKCCG Clubfoot andCovid-19.pdf (Accessed March 9, 2024).

[7] British Society for Children’s Orthopaedic Surgery. UK Clubfoot Consensus Group Guidance on Recovery of Clubfoot Services During the COVID-19 Pandemic. Available online at: www.bscos.org.uk/covid19/resources.php?sldownload=Y292aWQvUmVjb3Zlcnkgb2YgY2×1YmZvb3Qgc2VydmljZXMgZHVyaW5nIENvdmlkIDE5LnBkZiwwLCwsLDEsMTU4OTIxMjU3MiwxNDEzYzRlMWFjOTZlOWFkNDY3NTU4NmYwMmJkY2RjYg%3D%3D/Recovery%20of%20clubfoot%20services%20during%20Covid%2019.pdf (Accessed March 9, 2024).

[8] Bor N Herzenberg JE Frick SL. Ponseti management of clubfoot in older infants. Clin Orthop Relat Res. (2006) 444:224–8. 10.1097/01.blo.0000201147.12292.6b 16456307

[9] British Society for Children’s Orthopaedic Surgery. Removing Ponseti Casts Without Using the Plaster Saw. Available online at: www.bscos.org.uk/covid19/resources.php?sldownload=Y292aWQvUmVtb3ZpbmcgUG9uc2V0aSBDYXN0cy5wZGYsMCwsLCwxLDE1ODc5OTI3NDIsOWU1ZjYwZjk4NmY3YTNhYjE3ZWVlYjQxODdmYjRjOTc%3D/Removing Ponseti Casts.pdf (Accessed March 9, 2024).

[10] British Orthopaedic Association. British Orthopaedic Association casting committee guidance for casting practice in the current COVID-19 pandemic. Published 30 April 2020. Version 1. Available online at: https://www.boa.ac.uk/uploads/assets/f49aea2c-89e9-436b-8ac061e0dee079fe/Guidance-for-casting-practice-in-the-current-Covid-19-pandemic-FINAL.pdf (Accessed February 28, 2024).

[11] Morcuende JA Abbasi D Dolan LA Ponseti IV. Results of an accelerated ponseti protocol for clubfoot. J Pediatr Orthop. (2005) 25(5):623–6. 10.1097/01.bpo.0000162015.44865.5e 16199943

[12] Böhm S Sinclair M. Report of the 1st European consensus meeting on ponseti clubfoot treatment: Karolinska Institutet Stockholm, July 6th 2012. J Child Orthop. (2013) 7(3):251–4. 10.1007/s11832-013-0497-4

[13] British Society for Children’s Orthopaedic Surgery. DRAFT NHS/PHE Guidelines for Management of DDH During the COVID-19 Pandemic (updated 19.4.20 but still in draft). (2020). Available online at: https://www.bscos.org.uk/covid19/resources.php?sldownload=Y292aWQvRERIIEd1aWRlbGluZXMgQ292aWQxOS5wZGYsMCwsLCwxLDE1ODkwMzU3MzcsNmNlMmJmYThmNTQ0MDY4ZWM1Y2JkYmMyM2U3Y2Q3MjM%3D/DDH%20Guidelines%20Covid19.pdf (Accessed March 28, 2024).

[14] Paediatric Orthopaedic Society of North America. Managing DDH During COVID-19 Pandemic. April 2020. Available online at: www.posna.org/Blogs/POSNA-COVID-19-Updates/April-2020/Managing-DDH-During-COVID-19-Pandemic (Accessed February 20, 2024).

[15] Ömeroğlu H. Treatment of developmental dysplasia of the hip with the pavlik harness in children under six months of age: indications, results, and failures. J Child Orhop. (2018) 12:308–16. 10.1302/1863-2548.12.180055 30154920 PMC6090187

[16] Sankar WN Gornitzky AL Clarke NMP Herrera-Soto JA Kelley SP Matheney T Price CT; international hip dysplasia institute. Closed reduction for developmental dysplasia of the hip: early-term results from a prospective, multicenter cohort. J Pediatr Orthop. (2019) 39(3):111–8. 10.1097/BPO.0000000000000895 30730414 PMC6416015

[17] Upasani V Bomar JD Matheney TH Sankar WN Mulpuri K Price CT Evaluation of brace treatment for infant hip dislocation in a prospective cohort: defining the success rate and variables associated with failure. J Bone Joint Surg Am. (2016) 98(14):1215–21. 10.2106/JBJS.15.01018 27440570

[18] NHS England and NHS Improvement. Specialty guides for patient management during the coronavirus pandemic. Urgent and emergency musculoskeletal conditions in children (under 16) requiring onward referral. Publications approval reference: 001559. April 11 2020, Version 1. Available online at: www.england.nhs.uk/coronavirus/wp-content/uploads/sites/52/2020/04/C0289-specialty-guidance-emergency-and-urgent-msk-guidance-for-children.pdf (Accessed March 2, 2024).

[19] Australian Orthopaedic Association. AOA and subspecialty COVID-19 position statements and advisory documents pack. 28 April 2020. Available online at: www.aoa.org.au/docs/default-source/covid-19-member-documents/aoa-and-subspecialty-covid-19-advice-pack.pdf?sfvrsn=d9d8dd04_8 (Accessed March 3, 2024).

[20] Farrell S Schaeffer EK Mulpuri K. Recommendations for the care of pediatric orthopedic patients during the COVID pandemic. J Am Acad Orthop Surg. (2020) 10:5435/JAAOS-D-20-00391. 10.5435/JAAOS-D-20-00391 32301817 PMC7197339

[21] Trisolino G Origo CE De Sanctis N Dibello D Farsetti P Gigante C Recommendations from the Italian Society of Pediatric Orthopaedics and Traumatology for the management of pediatric orthopedic patients during the COVID19 pandemic and post-pandemic period in Italy. Ital J Pediatr. (2020) 46(1):149. 10.1186/s13052-020-00911-7 33032650 PMC7542566

[22] Hearty T Maizels M Pring M Mazur J Liu R Sarwark J Orthopaedic resident preparedness for closed reduction and pinning of pediatric supracondylar fractures is improved by e-learning. A multisite randomized controlled study. J Bone Joint Surg Am. (2013) 95(17):e1261–7. 10.2106/JBJS.L.01065 24005210

[23] Agarwal-Harding KJ Chokotho LC Mkandawire NC Martin C Jr Losina E Katz JN. Risk factors for delayed presentation among patients with musculoskeletal injuries in Malawi. J Bone Joint Surg Am. (2019) 101(10):920–31. 10.2106/JBJS.18.00516 31094984 PMC6530973

[24] NHS England and NHS Improvement. Specialty guides for patient management during the coronavirus pandemic. Clinical guide to surgical prioritisation during the coronavirus pandemic. Publications approval reference: 001559. April 11 2020 Version 1. Available online at: www.england.nhs.uk/coronavirus/wp-content/uploads/sites/52/2020/03/C0221-specialty-guide-surgical-prioritisation-v1.pdf (Accessed March 23, 2024).

[25] Swarup I LaValva S Shah R Sankar WN. Septic arthritis of the hip in children: a critical analysis review. JBJS Rev. (2020) 8(2):e0103. 10.2106/JBJS.RVW.19.00103 32224630

[26] American College of Surgeons. COVID-19 Guidelines for Triage of Pediatric Patients. 24 March 2020. Available online at: www.facs.org/covid-19/clinical-guidance/elective-case/pediatric-surgery (Accessed March 20, 2024).

[27] British Orthopaedic Association. British Orthopaedic Association Standards for Trauma. Management of patients with urgent orthopedic conditions and trauma during the coronavirus pandemic. Available online at: www.boa.ac.uk/uploads/assets/ee39d8a8-9457-4533-9774e973c835246d/4e3170c2-d85f-4162-a32500f54b1e3b1f/COVID-19-BOASTs-Combined-FINAL.pdf (Accessed January 9, 2024).

[28] NHS Greater Glasgow and Clyde. Royal Hospital for Children (RHC) Practical Fracture Management Guidance for use during COVID-19 crisis. Available online at: https://www.clinicalguidelines.scot.nhs.uk/ggc-paediatric-guidelines/ggc-guidelines/emergency-medicine/rhc-practical-fracture-management-guidance-for-use-during-covid-19-crisis (Accessed January 21, 2024).

[29] British Orthopaedic Association. British Orthopaedic Association Standards for Trauma. Supracondylar Fractures of the Humerus in Children. Available online at: www.boa.ac.uk/uploads/assets/8c4d6309-7428-4a88-ad1e485f5a66f33f/a71360df-cd0c-413a-b435f983b19957ee/supracondylar%20fractures%20of%20the%20humerus%20in%20children.pdf (Accessed January 29, 2024).

[30] Brizola E Adami G Baroncelli GI Bedeschi MF Berardi P Boero S Providing high-quality care remotely to patients with rare bone diseases during COVID-19 pandemic. Orphanet J Rare Dis. (2020) 15:228. 10.1186/s13023-020-01513-6 32867855 PMC7456755

[31] Sangiorgi L Brizola E, COVID-19 Helpline for Rare Bone Diseases Group. The line between COVID-19 pandemic and rare bone diseases. Ir J Med Sci. (2020) 190(3):1243–4. 10.1007/s11845-020-02400-6 33140293 PMC7605471

